# Inconsistent response of bacterial phyla diversity and abundance to soil salinity in a Chinese delta

**DOI:** 10.1038/s41598-021-92502-7

**Published:** 2021-06-18

**Authors:** Chao Yang, Kangjia Li, Dantong Lv, Shenyi Jiang, Junqi Sun, Hao Lin, Juan Sun

**Affiliations:** Grassland Agri-Husbandry Research Center, College of Grassland Science, Qingdao Agricultural University, Qingdao, 266109 China

**Keywords:** Microbial ecology, Environmental microbiology

## Abstract

Soil salinization is an increasingly serious problem and decreases crop yields in the Yellow River Delta (YRD), but its effects on bacterial community and diversity at the phylum level are not well known. We used high-throughput sequencing of soil bacterial 16S rRNA to identify soil bacterial communities and diversity across a gradient of soil salinity (electrical conductivity), namely, S1: low salinity level (1.78 ds/m), S2: medium salinity level (3.16 ds/m), S3: high salinity level (17.26 ds/m), S4: extreme salinity level (34.41 ds/m), and a non-salted site as the control (CK, 0.92 ds/m). Our results indicated the significantly higher values of soil C/N ratio in S2, S3, and S4 compared with that in CK. Significantly lower values of the Shannon and Chao 1 indexes were observed in S4 compared with the CK (*p* < 0.05). High salinity decreased the relative abundance of *Actinobacteria* and *Acidobacteria*, but increased that of *Gemmatimonadetes* and *Bacteroidetes*. Additionally, the Shannon diversity of *Bacteroidetes* increased by 15.5% in S4 compared with that in the CK. Our results indicate that soil salt is a main factor regulating bacterial phyla diversity and community in the extremely saline-alkaline soils of YRD. The high abundance and diversity of *Bacteroidetes* can be used for saline-alkali land restoration.

## Introduction

Soil salinization is considered one of the most pressing environmental challenges^1^, and more than 100 countries are estimated to be affected by salinization and alkalization^2^. The Yellow River Delta (YRD), located in Bohai Bay, is a regressive area formed by land-sea interactions in the northern part of Shandong Province, China^3^. Soil salinization in the YRD has spread at an unprecedented rate from shoreline to inland over the past 20 years^4^. The high soil salt content is the main limiting factor that not only affects plant growth^5^, but also influences soil microbial activities in this region.

Changes in salinity could lead to a decrease or an increase in some bacterial communities^6^. For example, a previous study found that *Betaproteobacteria* was enriched in the most saline-alkali soils^7^; however, the classes *Gammaproteobacteria* and *Alphaproteobacteria* remained unchanged. Numerous studies suggested that *Bacteroidetes* is the dominant phylum in saline-alkali soils^8,9^. The microbial response to soil salinity in the YRD has rarely been assessed until recently, and the *Bacteroidetes* and *Gemmatimonadetes* were enriched in high-salinity soils^4^. However, this result was only based on a narrow range of electrical conductivity from 0.34 ds/m to 6.73 ds/m^4^, it is necessary to explore the response of soil microorganisms to soil salinity in a wide range. In addition, studies on bacterial diversity at the phylum level in saline-alkali soils remain obscure, and thus we do not know whether the response of the main bacterial phyla diversity to soil salinity is consistent with that of bacterial abundance.

Many studies reported pH as the driver of soil bacterial communities across North and South America^10,11^, in alkaline lake sediments across the Qinghai Tibet Plateau^12^, in Changbai Mountain soils^13^, and in the saline-alkali soils of northeastern China^14^. However, a meta-analysis showed that the global soil microbial diversity and composition in saline soil are more strongly affected by salinity than by other extreme soil factors, such as pH^15^. Recently, Zhao et al.^4^ suggested a low impact of pH on bacterial community structure and diversity in saline-alkali lands. Hence, it is necessary to further compare the factors that have a strong impact on the structure and diversity of soil bacteria, especially in the severe salinization region of YRD.

To date, alterations in bacterial communities, determined using high-throughput sequencing methods, in saline-alkali soils of YRD have rarely been reported. In the present study, we measured the soil bacterial composition under five different salinity levels using high-throughput sequencing technology, and the bacterial diversity at the phylum level was also calculated. The objectives of the present study were to (1) determine the bacterial diversity and community at the phylum level along a wide range of salinity gradients, (2) identify the microbial groups with strong salt tolerance to saline-alkali soils, and (3) evaluate the key factors affecting soil bacterial diversity and community structure in the YRD.

## Results

### Soil bacterial responses to salinity

The soil salt, EC, and BD values significantly increased, while significantly lower values of TC were observed as the salinization level increased (Table S1; *p* < 0.05). The α-diversity of soil bacteria had a consistent response to soil salinity. S2 showed higher Sobs, ACE, and Chao 1 indices than CK, S1, S3, and S4. Significantly lower values of Sobs, Shannon, ACE, and Chao 1 indices were observed in S4 (Table 1; *p* < 0.05).

**Table 1:** The α-diversity and species richness of soil bacteria in the control (CK), low salinity level (S1), medium salinity level (S2), high salinity level (S3), and extreme salinity level (S4) sites.

	Sobs	Shannon	ACE	Chao1	Coverage (%)
CK	2548(58)b	6.50(0.03)a	3408(33)b	3427(43)b	97.4(0.3)b
S1	2543(68)b	6.45(0.08)a	3331(115)b	3312(105)b	97.7(0.2)b
S2	3052(131)a	6.55(0.06)a	4029(134)a	4004(125)a	97.5(0.1)b
S3	2616(44)b	6.07(0.03)b	3636(69)b	3569(44)b	97.8(0.2)b
S4	1301(176)c	5.15(0.06)c	1530(134)c	1485(126)c	99.3(0.2)a

Values are the mean ± standard error.

Sobs: the actual observed richness; Shannon: the Shannon diversity index; ACE: Ace index of species richness; Chao1: Chao1 index of species richness. In the table, the significant relationships at *p* < 0.05 are indicated by different letters based on the DUNCAN test.

**Figure 1:**
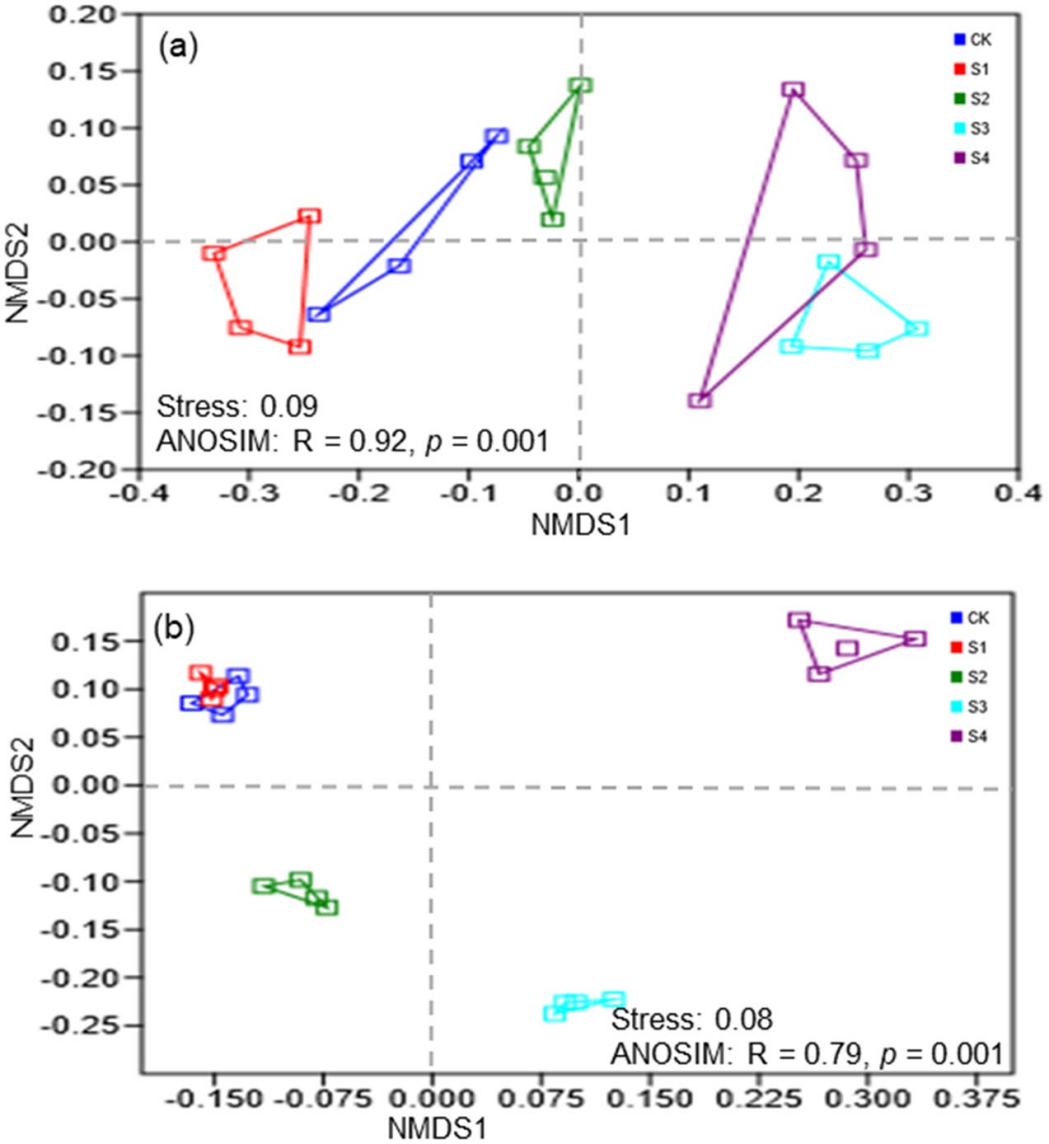
The NMDS ordinations based on the relative abundance of the bacterial communities (**a**) and Shannon diversity (**b**) of the soil bacterial phyla under five salinity levels.

**Figure 2:**
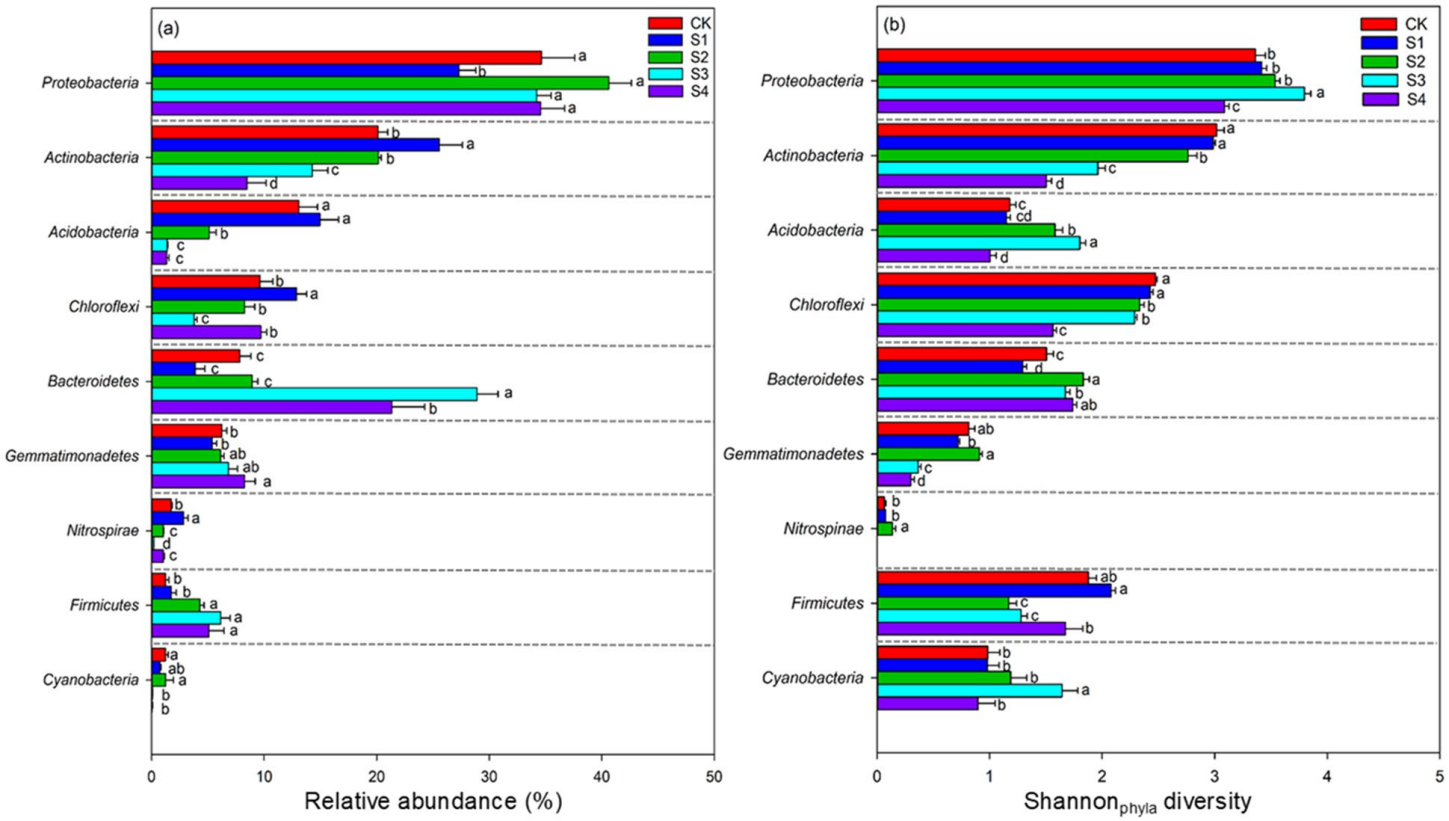
The relative abundances (**a**) and Shannon diversity (**b**) of the soil bacterial phyla under five salinity levels; significant relationships at *p* < 0.05 are indicated by different letters based on the DUNCAN test.

The NMDS and ANOSIM tests showed that the bacterial community composition in S2, S3, and S4 differed significantly from that in the CK at the OTU level (stress = 0.0001, R = 0.9596, *p* = 0.001; Fig. S1a). In addition, the NMDS and ANOSIM tests showed that the bacterial community composition in S1, S2, S3, and S4 differed significantly from that in the CK at the phylum level (stress = 0.09, R = 0.92, *p* = 0.001; Fig. 1a) and the class level (stress = 0.05, R = 0.90, *p* = 0.001; Fig. S2a), and the Shannon diversity at the phylum level in S1, S2, S3, and S4 also differed significantly from that in the CK (stress = 0.08, R = 0.79, *p* = 0.001; Fig. 1b). The phylum *Proteobacteria* occupied the largest proportion across the five salinity levels. Soil salinization significantly increased the relative abundances of *Gemmatimonadetes*, and S3 plot have the highest abundances of *Bacteroidetes* (Fig. 2a). However, the relative abundances of *Actinobacteria* and *Acidobacteria* under high salinity were lower than those under low salinity (Fig. 2a). Furthermore, the class *Alphaproteobacteria* and *Betaproteobacteria* under high salinity were lower than those under low salinity, and soil salinization significantly increased the relative abundances of *Gammaproteobacteria* (Fig. S2b).

The Shannon diversity at the phylum level is shown in Fig. 2b. Soil salinization significantly increased the Shannon diversity of *Bacteroidetes*. However, the Shannon diversity of *Actinobacteria* and *Chloroflexi* decreased dramatically at extremely high salinity (S4). Interestingly, the Shannon diversity of *Proteobacteria*, *Acidobacteria*, *Gemmatimonadetes* and *Cyanobacteria* showed an increase from CK to S3 and then a decrease as the salt increased from S3 to S4.

### Soil properties structuring the bacterial communities and diversity

**Figure 3:**
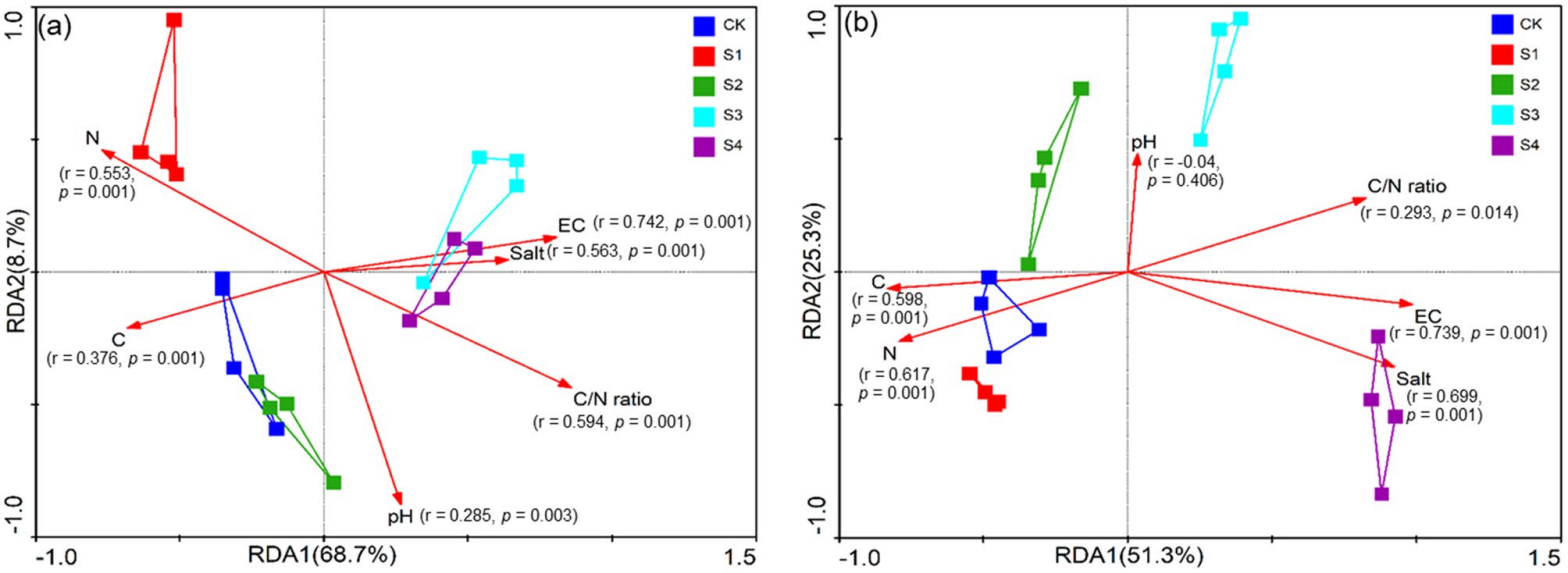
The RDA plots showing the effects of soil physiochemical properties (pH, EC, salt content, TC, TN, and C/N ratio) on the bacterial community structure at phylum level (**a**) and Shannon diversity (**b**). The significance of the effect of each property was examined using the Mantel test (permutation = 999), and the significance was evaluated by the r- and *p*-values.

The C/N ratio (r = 0.367, *p* = 0.003) and soil EC (r = 0.91, *p* = 0.001) were positively correlated with soil salt (r = 0.821, *p* = 0.001), which significantly affected the bacterial communities at the OTU level based on the Mantel test (Fig. S1b). The C/N ratio (r = 0.594, *p* = 0.001) and soil EC (r = 0.742, *p* = 0.001) were positively correlated with soil salt (r = 0.563, *p* = 0.001), which significantly affected the bacterial communities at the phylum level based on the Mantel test (Fig. 3a). At the class level, we also found that the soil salt (r = 0.718, *p* = 0.001), EC (r = 0.869, *p* = 0.001), C (r = 0.601, *p* = 0.001), N (r = 0.658, *p* = 0.001), and C/N ratio (r = 0.547, *p* = 0.001) significantly affected the bacterial communities (Fig. S2c). In addition, soil EC (r = 0.739, *p* = 0.001), soil salt (r = 0.699, *p* = 0.001), C (r = 0.598, *p* = 0.001), N (r = 0.617, *p* = 0.001), and the C/N ratio (r = 0.293, *p* = 0.014) significantly influenced the microbial Shannon diversity (Fig. 3b).

**Figure 4:**
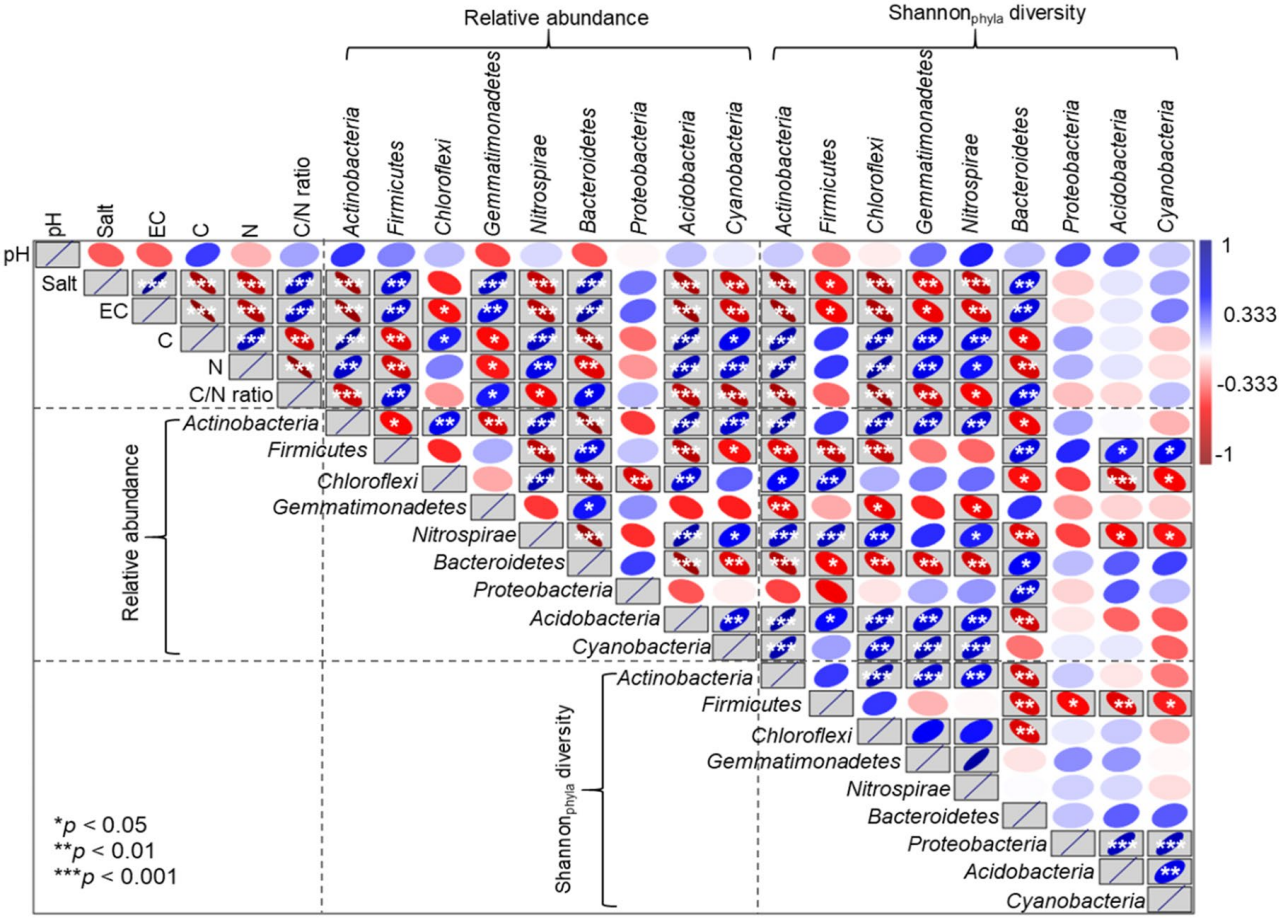
Spearman correlation analyses between soil physiochemical properties and the relative abundances and Shannon diversity of soil bacterial phyla. EC: electrical conductivity, TC: total carbon, TN: total nitrogen, and C/N ratio: soil total carbon/nitrogen. *, **, and *** indicate significance along the paths at *p* < 0.05, *p* < 0.01, and *p* < 0.001 levels, respectively.

Spearman correlation analyses showed that soil pH was not correlated with the bacterial abundance and diversity (Fig. 4). The relative abundances of *Acidobacteria*, *Actinobacteria*, *Nitrospirae*, and *Cyanobacteria* were negatively correlated with soil salt, EC, and the C/N ratio and positively correlated with soil C and N. In addition, The relative abundances of *Alphaproteobacteria* and *Betaproteobacteria* were negatively correlated with soil salt, EC, and the C/N ratio and positively correlated with soil C (Fig. S2d). However, the relative abundances of *Firmicutes*, *Gemmatimonadetes*, and *Bacteroidetes* were positively correlated with soil salt, EC, and the C/N ratio and negatively correlated with soil C and N. Interestingly, only the *Bacteroidetes* Shannon diversity showed a positive correlation with soil salt, EC, and the C/N ratio compared with the other phyla (Fig. 4).

## Discussion

The bacterial community under the five salinity levels was mainly formed by *Proteobacteria*, *Actinobacteria*, *Bacteroidetes*, *Gemmatimonadetes*, *Acidobacteria*, and *Chloroflexi* in this study. The phylum *Proteobacteria* occupied the largest proportion across the five salinity levels due to its high growth rate and strong metabolic capacity^16^. The relative abundance of *Proteobacteria* slightly increased as salinity increased. Similar findings reported that the Shannon diversity of *Proteobacteria* increased from CK to S3 and then declined significantly as salinity increased from S3 to S4^4^. Moreover, previous studies have also reported that the relative abundances of *Alphaproteobacteria* and *Gammaproteobacteria* were enriched in high-salinity soils^17^, and Gammaproteobacteria was also the dominant class in the saline soils in the salt lake system^18^, while *Betaproteobacteria* were more abundant in low-salinity soils^4^. Similar results were obtained also in the present study. Those results indicated that different bacterial species show different levels of tolerance to salt, even under the same phylum. The abundance of *Betaproteobacteria* decline in high-salinity soils due to the increase of the osmolarity outside the microbial cell^1^, while *Gammaproteobacteria* was reported to be halotolerant^19^, and the salt tolerance mechanism needs further study. Furthermore, the relative abundances of *Actinobacteria*, *Nitrospirae*, *Acidobacteria*, and *Chloroflexi* were negatively correlated with soil salinity. In particular, both *Actinobacteria* and *Acidobacteria* sharply declined with increasing soil salinity in this study. These bacteria generally prefer to live in acidic soils^4,20^.

Importantly, the phyla *Gemmatimonadetes* and *Bacteroidetes* are the two dominant populations in alkaline-saline soils and increased significantly as salinity increased in this study. In our previous study with higher salinity between pH 8.27 and 10.37 in semi-arid grassland, soil salinization dramatically increased the relative abundance of Gemmatimonadetes and Bacteroidetes^21^. Similarly, the relative abundance of the phylum *Gemmatimonadetes* increased following irrigation with salt water in irrigation system in arid desert^22^. However, some recent studies found that the abundance of *Bacteroidetes* was slightly affected by soil salinity in the YRD, which might be because this phylum can tolerate a wide salinity range^23,24^. *Bacteroidetes* showed no significant changes across a range of salinity levels from 0.34 to 6.73 ds m^−1^^4^. Interestingly, as the salinity levels increased from 0.92 to 34.41 ds m^−1^ in this study, the phylum *Bacteroidetes* was found to be significantly enriched in the extremely saline-alkaline soils. Additionally, the Shannon diversity of *Bacteroidetes* significantly increased in highly saline-alkaline soils. The salt tolerance mechanism and biological function of these microorganisms need further study in order to the ecological restoration in the extremely saline-alkaline soils of the YRD.

Salinity is commonly found to negatively influence the community structure and diversity of microbes in soils from reclaimed coastal land^25^ and coastal wetlands^16^. Salt stress increases the extracellular osmotic pressure of the microbial cell wall, decreasing microbial activities^1^. Indeed, RDA and the Mantel test revealed that soil salinity significantly affected the bacterial community structure and Shannon diversity.

A large part of the variation in soil bacterial composition is commonly related to differences in soil properties, such as the pH value^21^, salinity value^3^, ion concentrations^4^, and soil nutrient levels^26^. Generally, nutrient availability, serving as energy sources for microbes, is known to positively determine bacterial activities^27^. In the present study, some bacteria (*Firmicutes*, *Gemmatimonadetes*, and *Bacteroidetes*) were negatively correlated with soil C and N, and the relative abundances of *Acidobacteria*, *Actinobacteria*, *Nitrospirae*, and *Cyanobacteria* were positively correlated with soil C and N. As a recently emerged coastal wetland, nutrient contents (such as TN or TC) in the YRD were proved lower than in other coastal wetland sediments^28^. Along with soil salinity, TN and TC were significantly reduced, which affected distribution patterns of bacterial communities in this area. A study that conducted large-scale sampling of French soils indicated that pH was the dominant environmental factor controlling the bacterial phyla distribution^26^. A meta-analysis showed that the global microbial diversity and composition in saline soil are more affected by salinity than other extreme soil factors, such as pH^15^. Our recent findings revealed that soil salinity and pH have clearly defined the microbial communities in the high-salinity soils of the grasslands in northern China^21^, and the results of another study implied that soil pH is an equally important factor as soil salinity in shaping the soil bacterial community structure^18^. The RDA, Monte Carlo permutation test, and Spearman correlations in the current study suggested that soil pH had no effect on the bacterial abundance and diversity. Conversely, the soil salt content or EC was found to significantly influence the community structure and diversity of microbes. This is in accordance with the finding that soil salt is more important than soil pH in driving the bacterial phyla distribution in saline-alkaline soils of the YRD^4^. The soil salinity and pH commonly have a collinear relationship in saline-alkaline soils^18^; however, the soil salinity did not differ in response to soil pH based on Spearman correlation analyses in the current study. We speculate that soil bacteria in northeastern China are mainly affected by soil pH^21^, compared with salinity (soil EC and salt content) in the YRD. Hence, it is also important to study the relationship between soil saline and alkali conditions, especially in the extremely saline-alkaline soils of the YRD.

## Conclusions

This study explored the distribution patterns of soil bacterial communities and diversity in the extremely saline-alkaline soils of YRD. The α-diversity of soil bacteria showed a consistent response to soil salinity, and significantly lower values of the Shannon and Chao 1 indexes were observed under high salinity conditions. Bacterial diversity at the phylum level showed an inconsistent response to soil salinity compared with abundance. Compared with soil pH, the soil salt content is extremely negatively correlated with bacterial community and Shannon diversity, implying that soil salt was the main factor that shaped the soil bacterial communities and diversity in the extremely saline-alkaline soils of YRD. Considering its high salt tolerance, it is urgent to study the function of *Bacteroidetes* in order to the improvement and restoration of saline-alkali land. Our results provides a framework for future research to deeply analyze the mechanism and function of salt tolerance of soil bacteria in saline-alkaline environments.

## Materials and methods

### Study sites

**Figure 5:**
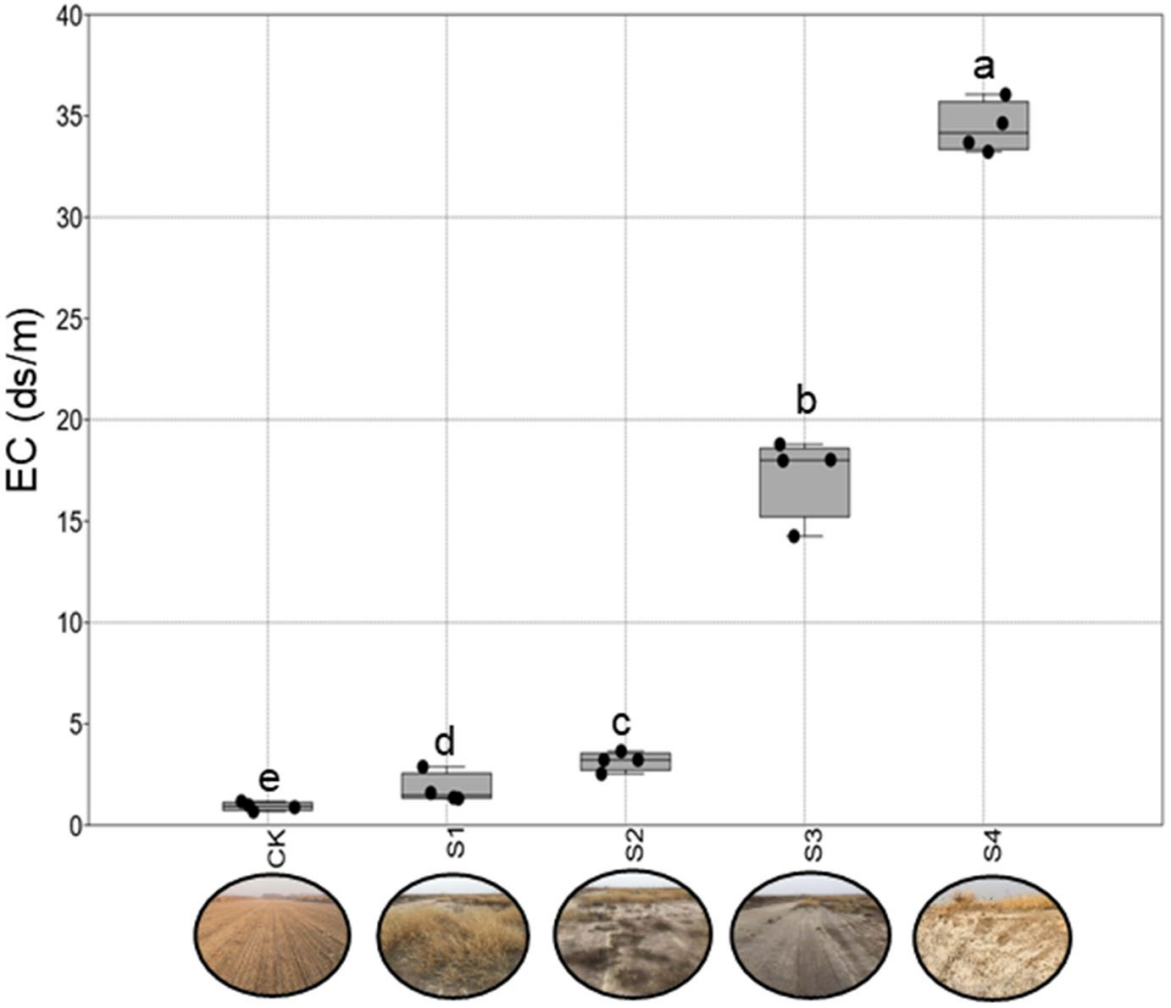
The salinization degree of sampling sites. CK: non-salted, S1: low salinity level, S2: medium salinity level, S3: high salinity level, S4: extreme salinity level. The photographs were taken by Chao Yang on October 2019. Significant relationships at *p* < 0.05 are indicated by different letters based on the DUNCAN test.

This study was located in Wudi County, which is part of the YRD in northern Shandong on the southern shore of the Bohai Sea (37°54′60″N, 117°57′33″E, elevation 1 m). This area has a semi-humid continental climate characterized by a mean annual precipitation and air temperature of 600 mm and 12 °C, respectively. We selected five different salinity levels from low to extreme salinization^29^. In brief, maize croplands with low salinity were selected as the control (CK) and are mainly affected by freshwater flooding. Land covered by *Setaria viridis*, low salt-tolerant vegetation, was selected as low salinity level (S1). Saline-alkali land dominated by *Suaeda salsa*, medium salt-tolerant vegetation, was selected as medium salinity level (S2). Saline-alkali land without vegetation growth but with salt crystallization was selected as high salinity level (S3), and extreme salinity level (S4) was the saline-alkali land with salt crystallization. The soil electrical conductivity value ranged from 0.92 ds/m (CK) to 1.78 ds/m (S1), 3.16 ds/m (S2), 17.26 ds/m (S3), and finally 34.41 ds/m (S4) according to our previous study (Fig. 5)^29^.

### Soil collection

Four transects across a distance of approximately 3 km represented four repetitions, and in each transect, five plots (CK, S1, S2, S3, and S4), which were spaced at least 500 m part, were randomly selected. In each plot (5 × 5 m^2^), the topsoil (0–15 cm) was collected using five-spot sampling in October 2019. The plant debris was removed, and we mix the five-point sample into one sample. Hence, a total of 20 samples (5 salinity levels × 4 repetitions) were collected, and we divided the soil samples into two subsamples. One subsample was air-dried for the analysis of basic soil properties, and the other part was placed in a − 80℃ freezer for microbiological analysis^21^. The physical and chemical properties and measurement methods of the soil are listed in the Supplementary Materials. Some basic characteristics for the soils in Table S1 were cited from our previous study^29^.

### High-throughput sequencing of soil bacteria

The genomic DNA was extracted from 0.30 g of soil using the MoBio PowerSoil DNA Isolation Kit (QIAGEN, Ins., USA). The V3-V4 regions of the bacterial 16S rRNA gene were amplified using universal primers 338F (5’-ACTCCTACGGGAGGCAGCAG-3’) and 806R (5’-GGACTACHVGGGTWTCTAAT-3’)^30^. The PCR analysis included pre-denaturation at 95 °C for 3 min, 27 cycles at 95 °C for 30 s, annealing at 55 °C for 30 s, elongation at 72 °C for 45 s, and an extension at 72 °C for 10 min.

Illumina MiSeq sequencing produced double-ended sequence data (2 × 300) according to standard protocols performed by MajorBio Bio-Pharm Technology Co. Ltd. (Shanghai, China). The obtained sequences were first filtered using the quantitative insights into microbial ecology. Raw FASTQ files were de-multiplexed and quality-filtered with the following criteria: (i) 300-bp reads were truncated at any site with an average quality score < 20 over a 50-bp sliding window, and truncated reads shorter than 50 bp were discarded; (ii) exact barcode matching, less than two nucleotide mismatches in the primer, and reads containing ambiguous bases were removed; (iii) only overlapping sequences longer than 10 bp were assembled according to their overlapped sequence. Reads that could not be assembled were discarded. Then, we used UPARSE ver. 7.1 to cluster the high-quality sequences with 97% identity threshold into operational taxonomic units (OTUs)^31^. The chimeric was identified and removed using UCHIME., and the chloroplasts and mitochondria sequences were removed at the classification step using Greengenes database. Because the sequencing depth varied across samples, we used a sub-sampling procedure to normalize the number of reads to the minimum observed across all samples. In total, 1,145,774 raw read sequences were obtained from 20 soil samples with at least 32,212 sequences per sample. A total of 48,240 observed OTUs were obtained from all samples with an average of 2,412 per sample.

### Statistical analysis

The α-diversity indices, including the coverage, Sobs (the actual observed richness), ACE (Ace index of species richness), Chao1 (Chao1 index of species richness), and Shannon diversity index, were classified using MOTHUR software. The Shannon diversity at the phylum level was calculated as follows^21^:

$$ {\text{Shannon}}_{{{\text{phyla}}}}  =  - \sum \left( {\frac{{{\text{Ni}}}}{{\text{N}}}} \right)\ln \left( {\frac{{{\text{Ni}}}}{{\text{N}}}} \right), $$

where Shannon _phyla_ is the bacterial diversity at the phylum level, *N* is the total number of OTUs in each bacterial phylum, and *Ni* is the number of individuals in group *i*.

The significant differences in the soil total bacterial α-diversity, bacterial Shannon diversity at the phylum level, bacterial community, and soil physicochemical properties of the five salinization levels were evaluated using one-way ANOVA in SPSS (ver. 19.0). The significance was analyzed at *p* < 0.05 using DUNCAN’s test.

Nonmetric multidimensional scaling (NMDS) based on Bray–Curtis similarity matrices was performed to identify the response of soil bacteria to salinity. The significance was tested by analysis of similarities (ANOSIM) in PAST (ver. 3.25).

The relationships between soil physicochemical properties and the soil bacterial communities at the phylum and class level and the diversity of bacterial phyla were analyzed by redundancy analysis (RDA) using CANOCO (ver. 4.5). The significance of the effect of each property was examined using the Mantel test (permutation = 999), and the significance was evaluated by the r- and *p*-values. Spearman analyses were performed to identify the correlations between the soil physicochemical properties and the relative abundances and diversity of bacterial phyla.

## Supplementary Information

Supplementary Information.

## Data Availability

Sequence data supporting the findings of this study have been deposited at NCBI database under Sequence Read Archive (SRA) accession number SRP268965.

## References

[1] Rath KM, Rousk J (2015). Salt effects on the soil microbial decomposer community and their role in organic carbon cycling: a review. Soil Biol. Biochem..

[2] Rengasamy P (2006). World salinization with emphasis on Australia. J. Exp. Bot..

[3] Guan B (2020). Salt is a main factor shaping community composition of arbuscular mycorrhizal fungi along a vegetation successional series in the Yellow River Delta. CATENA.

[4] Zhao Q (2020). Shifts of soil bacterial community along a salinity gradient in the Yellow River Delta. Land Degrad. Dev..

[5] Cui B, Yang Q, Yang Z, Zhang K (2009). Evaluating the ecological performance of wetland restoration in the Yellow River Delta, China. Ecol. Eng..

[6] Rath KM, Maheshwari A, Rousk J (2019). Linking Microbial Community Structure to Trait Distributions and Functions Using Salinity as an Environmental Filter. MBio.

[7] Jiang H (2007). Microbial response to salinity change in Lake Chaka, a hypersaline lake on Tibetan plateau. Environ. Microbiol..

[8] Keshri J, Mody K, Jha B (2013). Bacterial community structure in a semi-arid haloalkaline soil using culture independent method. Geomicrobiol J..

[9] Zheng W (2017). The responses and adaptations of microbial communities to salinity in farmland soils: a molecular ecological network analysis. Appl. Soil. Ecol..

[10] Fierer N, Jackson RB (2006). The diversity and biogeography of soil bacterial communities. Proc. Natl. Acad. Sci. USA.

[11] Lauber CL, Hamady M, Knight R, Fierer N (2009). Pyrosequencing-based assessment of soil pH as a predictor of soil bacterial community structure at the continental scale. Appl. Environ. Microbiol..

[12] Xiong J (2012). Geographic distance and pH drive bacterial distribution in alkaline lake sediments across Tibetan Plateau. Environ. Microbiol..

[13] Shen C (2013). Soil pH drives the spatial distribution of bacterial communities along elevation on Changbai Mountain. Soil Biol. Biochem..

[14] Liu J (2014). High throughput sequencing analysis of biogeographical distribution of bacterial communities in the black soils of northeast China. Soil Biol. Biochem..

[15] Ma B, Gong J (2013). A meta-analysis of the publicly available bacterial and archaeal sequence diversity in saline soils. World J. Microbiol. Biotechnol..

[16] Behera P (2017). Salinity and macrophyte drive the biogeography of the sedimentary bacterial communities in a brackish water tropical coastal lagoon. Sci. Total Environ..

[17] Kawaichi S (2013). Ardenticatena maritima gen nov, sp nov, a ferric iron- and nitrate-reducing bacterium of the phylum 'Chloroflexi' isolated from an iron-rich coastal hydrothermal field, and description of Ardenticatenia classis nov. Int. J. Syst. Evol. Microbiol..

[18] Zhao S (2018). Soil pH is equally important as salinity in shaping bacterial communities in saline soils under halophytic vegetation. Sci. Rep..

[19] Zeglin LH (2011). Bacterial community structure along moisture gradients in the parafluvial sediments of two ephemeral desert streams. Microb. Ecol..

[20] Lv X (2014). A meta-analysis of the bacterial and archaeal diversity observed in wetland soils. Sci. World J..

[21] Yang C (2020). Assessing the effect of soil salinization on soil microbial respiration and diversities under incubation conditions. Appl. Soil. Ecol..

[22] Guo H, Hu Z, Zhang H, Hou Z, Min W (2019). Soil microbial metabolic activity and community structure in drip-irrigated calcareous soil as affected by irrigation water salinity. Water Air Soil Pollut..

[23] Riemann L (2008). The native bacterioplankton community in the central Baltic sea is influenced by freshwater bacterial species. Appl. Environ. Microbiol..

[24] Zhang L, Gao G, Tang X, Shao K, Gong Y (2016). Pyrosequencing analysis of bacterial communities in Lake Bosten, a large brackish inland lake in the arid northwest of China. Can. J. Microbiol..

[25] Kim K (2019). Structural and functional responses of microbial community with respect to salinity levels in a coastal reclamation land. Appl. Soil. Ecol..

[26] Karimi B (2018). Biogeography of soil bacteria and archaea across France. Sci. Adv..

[27] Francioli D, Schulz E, Buscot F, Reitz T (2018). Dynamics of soil bacterial communities over a vegetation season relate to both soil nutrient status and plant growth phenology. Microb. Ecol..

[28] Yu J (2016). Distribution of carbon, nitrogen and phosphorus in coastal wetland soil related land use in the Modern Yellow River Delta. Sci. Rep..

[29] Yang C, Sun J (2020). Soil salinity drives the distribution patterns and ecological functions of Fungi in Saline-Alkali land in the Yellow River Delta, China. Frontiers Microbiol..

[30] Li J, Yang C (2019). Inconsistent response of soil bacterial and fungal communities in aggregates to litter decomposition during short-term incubation. PeerJ.

[31] Caporaso JG (2012). Ultra-high-throughput microbial community analysis on the Illumina HiSeq and MiSeq platforms. ISME J..

