# Supplementary material for: Inconsistent response of bacterial phyla diversity and abundance to soil salinity in a Chinese delta
Source: Sci Rep. 2021 Jun 18;11:12870. doi: 10.1038/s41598-021-92502-7 (PMC8213812; doi:10.1038/s41598-021-92502-7)

**Inconsistent response of bacterial phyla diversity and abundance to soil salinity in a Chinese delta**

Chao Yang^1^; Kangjia Li ^1^; Dantong Lv ^1^; Shenyi Jiang^1^; Junqi Sun^1^; Hao Lin ^1^; Juan Sun ^1*^

^1^ College of Grassland Science, Qingdao Agricultural University, Qingdao 266109, China

^*^Correspondence author

Email: sunjuan@qau.edu.cn (Juan Sun)

**Appendix A Supplementary data**

**Processing of soil properties analysis**

Soil total carbon (TC) and soil total nitrogen (TN) concentrations were measured using a CHNS Element Analyser (Elementar, Germany). Soil pH and electrical conductivity (EC) were measured using a glass electrode in a 1: 2.5 soil: water suspension. The soil salt content was determined in a mixture with a soil : water ratio of 1:2.5, and the soil extract was then dried at 105°C for 24 h.

**Table S1** One-way ANOVA of the soil properties of non-salted (CK), low salinity level (S1), medium salinity level (S2), high salinity level (S3), extreme salinity level (S4) sites. Values are mean ± standard error.

|  | EC (ds m^-1^) | Salt (%) | pH | TC (g kg^-1^) | TN (g kg^-1^) | C/N ratio |
| --- | --- | --- | --- | --- | --- | --- |
| CK | 0.92(0.10)e | 0.04(0.01)d | 8.70(0.05)a | 21.10(1.13)a | 0.63(0.05)b | 34.70(4.59)d |
| S1 | 1.78(0.37)d | 0.16(0.05)c | 8.18(0.07)c | 17.40(0.50)b | 0.85(0.09)a | 21.11(2.12)e |
| S2 | 3.16(0.23)c | 0.18(0.01)c | 8.75(0.04)a | 16.35(1.17)b | 0.40(0.04)c | 41.59(3.20)c |
| S3 | 17.26(1.01)b | 0.88(0.09)b | 8.56(0.01)b | 14.33(0.50)c | 0.38(0.08)c | 47.96(1.69)b |
| S4 | 34.41(0.63)a | 3.58(0.13)a | 8.49(0.01)b | 10.78(0.11)d | 0.20(0.01)d | 53.88(0.55)a |

Note: EC (electrical conductivity), TC (soil total carbon), TN (soil total nitrogen), C/N ratio (soil total carbon to total nitrogen ratios). In the list, the significant relationships at *p* < 0.05 was indicated by different letters using the DUNCAN test.

**Fig. S1** The NMDS ordinations based on the relative abundance of the bacterial communities at the OTU level (a), and the RDA (b) showing the effects of soil physiochemical properties (pH, EC, salt content, TC, TN, and C/N ratio) on the bacterial community structure at OTU level.

**
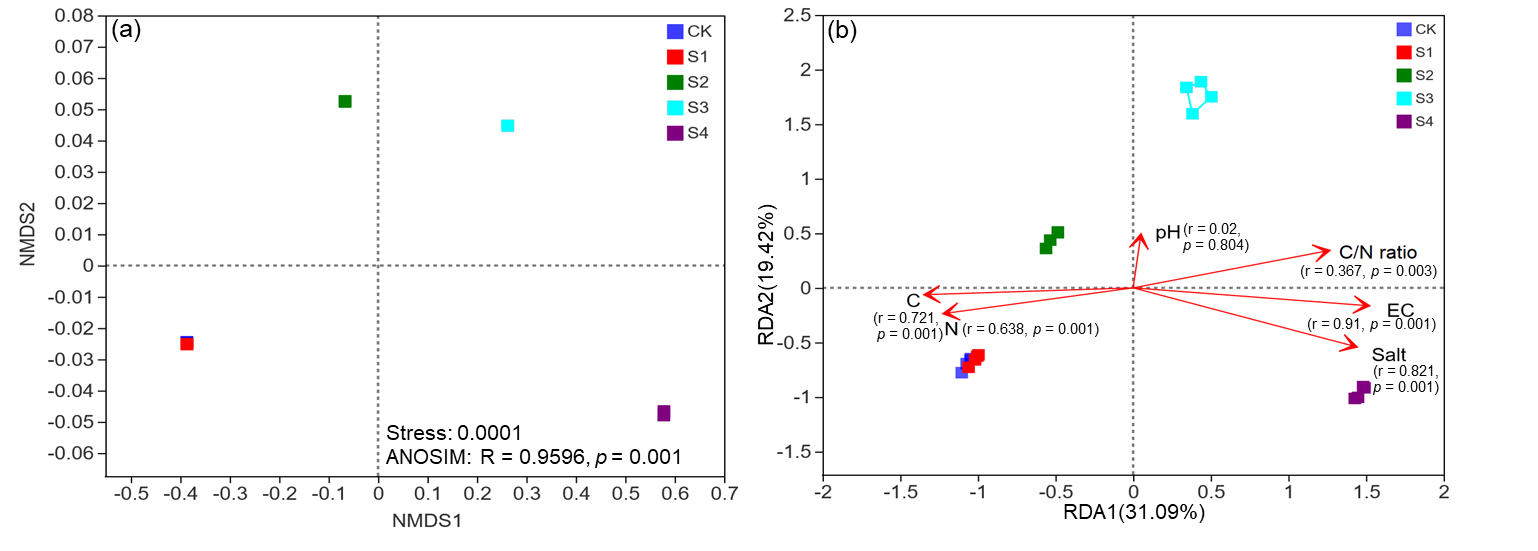
**

**Fig. S2** The NMDS ordinations based on the relative abundance of the bacterial communities at the class level (a), and the relative abundances (b) of the soil bacterial class under five salinity levels. The RDA (c) and Spearman correlation analyses (d) showing the effects of soil physiochemical properties (pH, EC, salt content, TC, TN, and C/N ratio) on the bacterial community structure at class level.


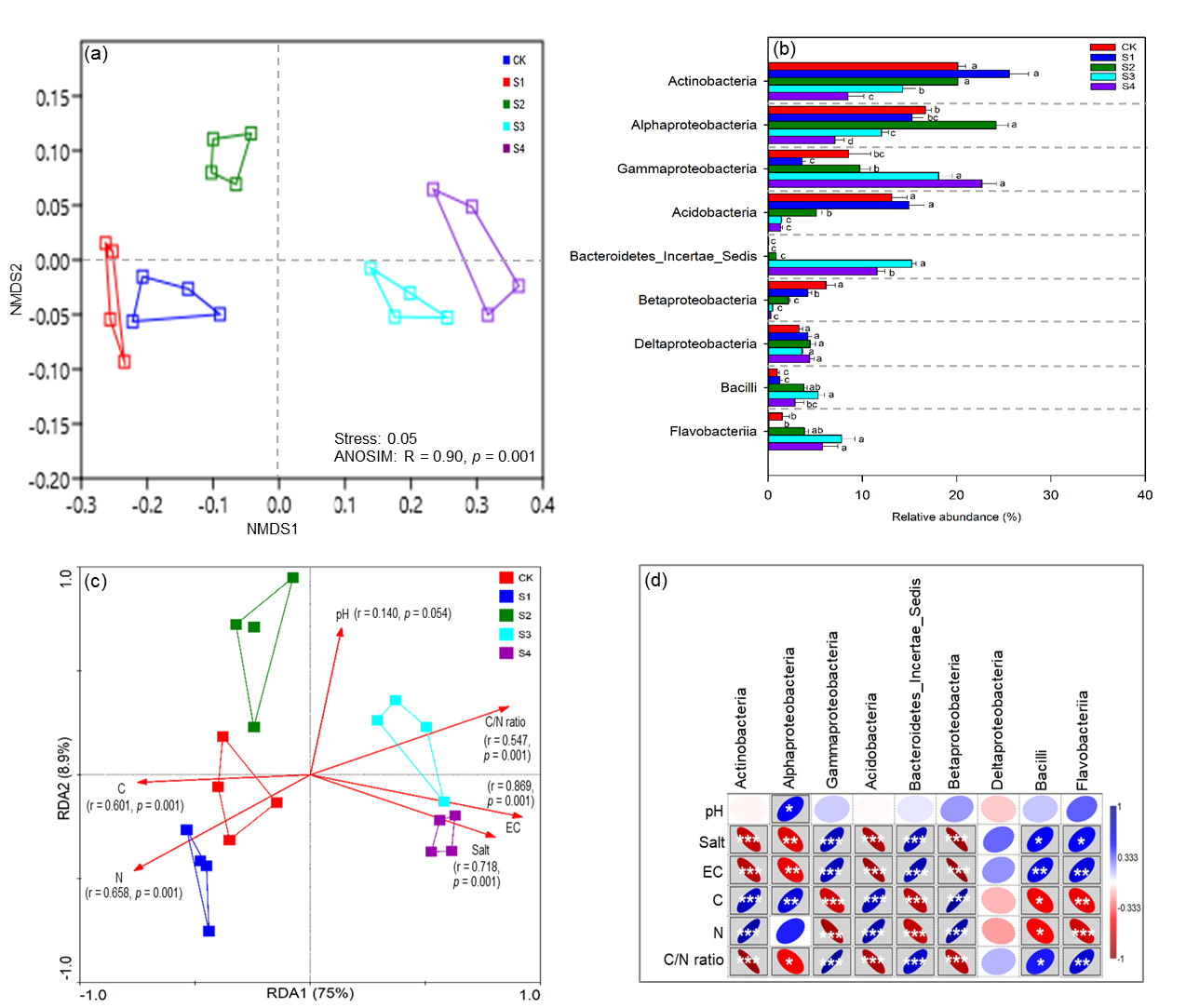

Supplement: Supplementary file 1 — Supplementary Information. [file 41598_2021_92502_MOESM1_ESM.docx]
